# Dephosphorylation of cGAS by PPP6C impairs its substrate binding activity and innate antiviral response

**DOI:** 10.1007/s13238-020-00729-3

**Published:** 2020-05-30

**Authors:** Mi Li, Hong-Bing Shu

**Affiliations:** Department of Infectious Diseases, Frontier Science Center for Immunology and Metabolism, Medical Research Institute, Zhongnan Hospital of Wuhan University, College of Life Sciences, Wuhan University, Wuhan, 430071 China

**Keywords:** DNA virus, PPP6C, cGAS, innate immune response, phosphorylation, substrate binding

## Abstract

The cyclic GMP-AMP (cGAMP) synthase (cGAS) plays a critical role in host defense by sensing cytosolic DNA derived from microbial pathogens or mis-located cellular DNA. Upon DNA binding, cGAS utilizes GTP and ATP as substrates to synthesize cGAMP, leading to MITA-mediated innate immune response. In this study, we identified the phosphatase PPP6C as a negative regulator of cGAS-mediated innate immune response. PPP6C is constitutively associated with cGAS in un-stimulated cells. DNA virus infection causes rapid disassociation of PPP6C from cGAS, resulting in phosphorylation of human cGAS S435 or mouse cGAS S420 in its catalytic pocket. Mutation of this serine residue of cGAS impairs its ability to synthesize cGAMP upon DNA virus infection. *In vitro* experiments indicate that S420-phosphorylated mcGAS has higher affinity to GTP and enzymatic activity. PPP6C-deficiency promotes innate immune response to DNA virus in various cells. Our findings suggest that PPP6C-mediated dephosphorylation of a catalytic pocket serine residue of cGAS impairs its substrate binding activity and innate immune response, which provides a mechanism for keeping the DNA sensor cGAS inactive in the absence of infection to avoid autoimmune response.

## Introduction

The innate immune system represents the first line of host defense against viral infection. Upon viral infection, structurally conserved viral components called pathogen associated molecular patterns (PAMPs) are detected by host pattern recognition receptors (PRRs), which triggers intracellular signaling events that lead to induction of type I interferons (IFNs), proinflammatory cytokines and other downstream effectors. These downstream effectors inhibit viral replication, facilitate clearance of virus-infected cells, and promote adaptive immune response (Janeway and Medzhitov, 2002; Akira et al., 2006; Hu and Shu, 2018).

Viral nucleic acids are major PAMPs recognized by host PRRs. Upon DNA virus infection, viral DNA in the cytoplasm is sensed by cyclic GMP-AMP (cGAMP) synthase (cGAS). Importantly, cGAS also detects mitochondrial DNA and nuclear DNA mis-located in the cytosol under certain conditions, which plays a key role in certain autoimmune diseases and inflammatory responses during radiotherapy for cancer (West et al., 2015; Fang et al., 2016; Harding et al., 2017; Mackenzie et al., 2017). Upon binding to DNA, cGAS utilizes GTP and ATP as substrates to synthesize the second messenger 2’3’-cGAMP (Sun et al., 2013; Wu et al., 2013). Structural analysis indicates that cGAS is activated through conformational transitions upon DNA binding, which results in formation of a catalytically competent and nucleotide substrate accessible pocket for generation of 2’3’-cGAMP. Cyclization of 2’3’-cGAMP occurs in a stepwise manner through initial generation of GTP-AMP, which is positioned precisely in the catalytic pocket (Gao et al., 2013; Xia et al., 2016). After synthesis, cGAMP binds to and activates the ER membrane-associated adaptor MITA (also called STING and ERIS) (Ishikawa and Barber, 2008; Zhong et al., 2008; Sun et al., 2009). MITA is then translocated from the ER via Golgi apparatus to perinuclear punctate structures. In this process, MITA recruits TBK1 and IRF3, leading to phosphorylation and activation of IRF3, induction of downstream antiviral effectors genes, and innate antiviral response (Ishikawa and Barber, 2008; Zhong et al., 2008; Liu et al., 2015).

Although transient activation of the cGAS-MITA axis is essential for host defense to DNA pathogens or aberrant self-DNA, its de-regulation causes sever autoimmune diseases such as systemic lupus erythematosus (SLE), Aicardi-Goutières syndrome, and STING-associated vasculopathy with onset in infancy (SAVI) (Liu et al., 2014; Gray et al., 2015; Xia et al., 2016; An et al., 2017; Hu and Shu, 2019). Therefore, the cGAS-MITA axis is heavily regulated by various mechanisms, particularly intracellular organelle trafficking and post-translational modifications (Hu and Shu, 2018; Luo and Shu, 2018). For example, cGAS is regulated by serine phosphorylation, polyubiquitination, sumoylation and acetylation (Hu et al., 2016; Hu and Shu, 2018; Xiong et al., 2018; Dai et al., 2019). However, the exact mechanisms on how cGAS activity is regulated are largely unknown.

PPP6C is a catalytic subunit of the protein phosphatase 6 (PP6), which belongs to protein serine/threonine phosphatase (PSP) family. PSPs are ancient enzymes which are conserved across eukaryotic evolution (Brautigan and Shenolikar, 2018). Previously, it has been shown that members of PSPs play important roles in regulation of innate immune responses. PP1α and PP1γ have been reported to dephosphorylate MDA5 and RIG-I, leading to their activation and innate immune responses to RNA viruses (Wies et al., 2013). PP2A dephosphorylates IRF3, resulting in its inactivation upon being challenged with LPS, poly(I:C) or low-titer Sendai virus (SeV) (Long et al., 2014). PPP4C dephosphorylates TBK1 at S172 to inhibit its activation (Zhan et al., 2015). It has been shown that PPP6C negatively regulates IL-1 signaling via dephosphorylating TAK1 at T187 (Kajino et al., 2006). Previous studies have also reported that PP6 is required for homology-directed repair of DNA double-strand breaks (Zhong et al., 2011). Dys-regulated PP6 activity plays a role in melanoma development (Hodis et al., 2012) and PPP6C is a somatic driver gene in skin basal cell carcinoma (Bonilla et al., 2016). In this study, we found that PPP6C suppressed phosphorylation of human cGAS (hcGAS) at S435 or mouse cGAS (mcGAS) at S420 in its substrate-binding pocket, thus preventing its binding to GTP and inhibiting the synthesis of cGAMP. Deficiency of PPP6C promoted innate immune response to DNA virus in mice. Our findings suggest that PPP6C-mediated dephosphorylation of cGAS impairs its substrate binding activity and innate immune response, which is important to keep cGAS inactive in the absence of infection to avoid autoimmune responses.

## Results

### PPP6C negatively regulates innate immune response to DNA virus

**Figure 1:**
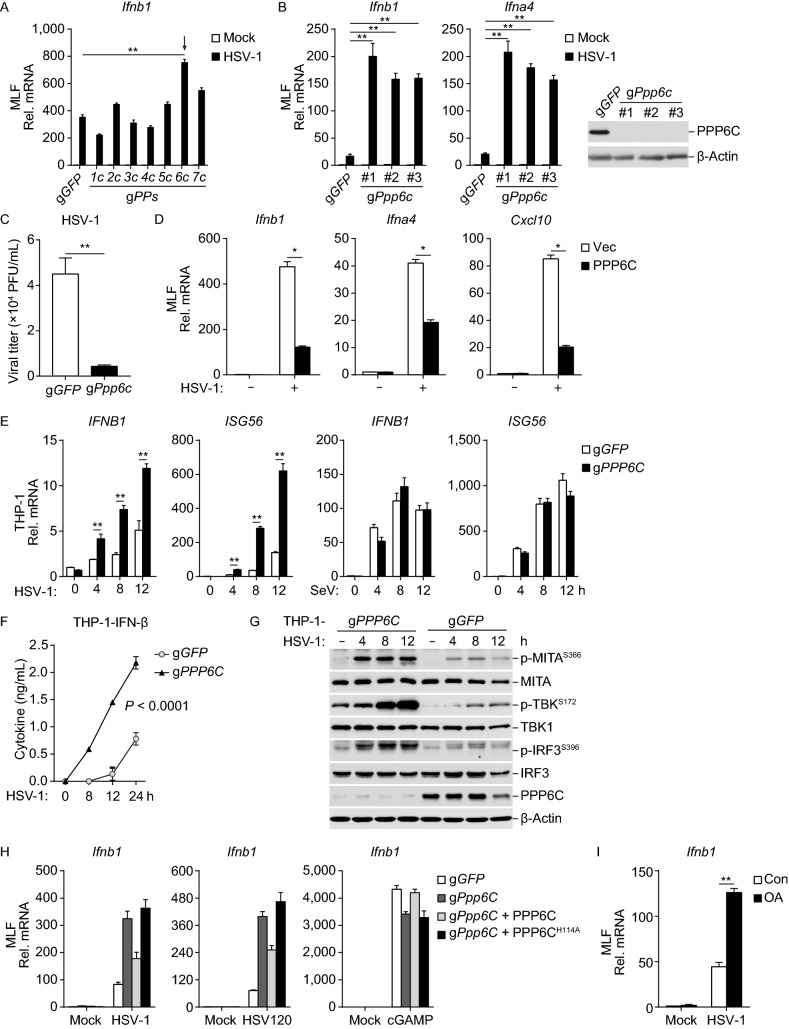
**PPP6C negatively regulates innate immune response to DNA virus**. (A) Screening of protein serine/threonine phosphatases that regulate HSV-1-induced transcription of *Ifnb1* gene. MLFs with CRISPR-Cas9-mediated knockout of the indicated genes (1 × 10^6^) were left untreated or infected with HSV-1 (MOI = 1) for 6 h before qPCR analysis. Graph shows mean ± SEM, *n* = 3. **P < 0.01. Data were analyzed using two-way ANOVA with Prism GRAGHPAD 7. (B) Deficiency of PPP6C enhances HSV-1-induced transcription of downstream genes in MLFs. The indicated control or three independent PPP6C-deficient MLFs (1 × 10^6^) were left untreated or infected with HSV-1 (MOI = 1) for 6 h before qPCR analysis of mRNA levels of the indicated genes. Graph shows mean ± SEM, *n* = 3. **P < 0.01. Data were analyzed using two-way ANOVA with Prism GRAGHPAD 7. The knockout efficiencies of PPP6C by the indicated gRNAs were analyzed by immunoblots. (C) Deficiency of PPP6C impairs HSV-1 replication. The control or PPP6C-deficient MLFs (1 × 10^6^) were infected with HSV-1 (MOI = 0.01) for 36 h before plaque assays. Graph shows mean ± SEM, *n* = 3. **P < 0.01. Data were analyzed using a Student’s unpaired *t*-test with Prism GRAGHPAD 7. (D) PPP6C inhibits HSV-1-induced transcription of downstream genes. The control or PPP6C-overexpressing MLFs (1 × 10^6^) were left untreated or infected with HSV-1 (MOI = 1) for 6 h before qPCR analysis for mRNA levels of the indicated genes. Graph shows mean ± SEM, *n* = 3. *P < 0.05. Data were analyzed using two-way ANOVA with Prism GRAGHPAD 7. (E) Deficiency of PPP6C enhances HSV-1-but not SeV-induced transcription of downstream genes in THP-1 cells. The control or PPP6C-deficient THP-1 cells (1 × 10^6^) were left untreated, infected with HSV-1 (MOI = 1) or infected with SeV (MOI = 1) for the indicated times before qPCR analysis of mRNA levels of the indicated genes. Graph shows mean ± SEM, *n* = 3. **P < 0.01. Data were analyzed using two-way ANOVA with Prism GRAGHPAD 7. (F) Deficiency of PPP6C increases secreted IFN-β level following HSV-1 infection. The control or PPP6C-deficient THP-1 cells (1 × 10^6^) were left untreated or infected with HSV-1 (MOI = 1) for the indicated times before the culture medium were collected for ELISA. Graph shows mean ± SEM, *n* = 3. **P < 0.01. Data were analyzed using two-way ANOVA with Prism GRAGHPAD 7. (G) Deficiency of PPP6C increases phosphorylation of MITA, TBK1 and IRF3. The control or PPP6C-deficient THP-1 cells (1 × 10^6^) were left untreated or infected with HSV-1 (MOI = 1) for the indicated times before immunoblot analysis with the indicated antibodies. (H) The phosphatase activity of PPP6C is essential for its inhibitory effects on HSV-1-induced signaling. The PPP6C-deficient MLFs reconstituted with PPP6C or PPP6C (H114A) (1 × 10^6^) were left untreated or treated with the indicated stimuli for 6 h before qPCR analysis of mRNA levels of the *Ifnb1* gene. (I) Okadaic acid (OA) inhibits HSV-1-induced transcription of *Ifnb1* gene. MLFs (1 × 10^6^) were left untreated or pre-treated with OA (100 nmol/L), then infected with HSV-1 (MOI = 1) for 6 h before qPCR analysis of mRNA levels of the *Ifnb1* gene. Graph shows mean ± SEM, *n* = 3. **P < 0.01. Data were analyzed using two-way ANOVA with Prism GRAGHPAD 7

Recently, it has been demonstrated that several key components of the cGAS-MITA signaling pathway, including cGAS, MITA, TBK1 and IRF3, are regulated by serine/threonine phosphorylation (Liu et al., 2015; Liu et al., 2018; Hu et al., 2019; Xia et al., 2019). Therefore, we attempted to identify protein phosphatases that regulate innate immune response to DNA virus. To do this, we mutated the protein serine/threonine phosphatases in SV40-immortalized murine lung fibroblasts (MLFs) by CRISPR-Cas9 system and examined the effects on transcription of interferon-1 (*Ifnb1*) gene induced by the DNA virus herpes simplex virus type 1 (HSV-1). These efforts led to the identification of PPP6C as a potential negative regulator in HSV-1-induced transcription of *Ifnb1* gene. As shown in Fig. 1A, knockout of PPP6C markedly promoted HSV-1-induced transcription of *Ifnb1* gene in MLFs. Further experiments indicated that knockout of PPP6C by each of the three independent PPP6C gRNAs enhanced HSV-1-induced transcription of *Ifnb1* and *Ifna4* genes in MLFs (Fig. 1B). The following experiments were performed with the #1 gRNA (g*Ppp6c*)-derived cells. In plaque assays, deficiency of PPP6C inhibited replication of HSV-1 in MLFs (Fig. 1C). Further experiments indicated that overexpression of PPP6C inhibited HSV-1-induced transcription of downstream antiviral genes in MLFs (Fig. 1D). To determine whether PPP6C functions in a species and cell specific manner, we constructed PPP6C-deficient human monocytic THP-1 cells by the CRISPR-Cas9 method for further experiments. These experiments revealed that deficiency of PPP6C enhanced HSV-1- but not the RNA virus Sendai virus (SeV)-induced transcription of *IFNB1* and *ISG56* genes (Fig. 1E). PPP6C-deficiency also inhibited HSV-1-induced secretion of IFN-β cytokine in the medium (Fig. 1F). Additionally, HSV-1-induced phosphorylation of MITA, TBK1 and IRF3 was markedly enhanced in PPP6C-deficient THP-1 cells (Fig. 1G). These data suggest that PPP6C negatively regulates innate antiviral response to DNA virus in both mouse and human cells.

Since human and murine PPP6C are 99% identical in amino acid sequence, we reconstituted the PPP6C-deficient murine MLFs with human wild-type PPP6C or PPP6C (H114A), an inactive mutant in which Histidine (H) 114 is mutated to alanine (A) (Hosing et al., 2012). qPCR experiments indicated that reconstituted wild-type PPP6C but not PPP6C (H114A) inhibited transcription of *Ifnb1* gene triggered by HSV-1 infection, or transfection of 120-mer DNA representing HSV-1 genome (HSV120) but not 2’3’-cGAMP in MLFs (Fig. 1H). Consistently, Okadaic acid (OA), an inhibitor of PSPs, enhanced HSV-1-induced transcription of *Ifnb1* gene in MLFs (Fig. 1I). These results suggest that PPP6C-mediated negative regulation of DNA-triggered innate immune response is dependent on its phosphatase activity.

**Figure 2:**
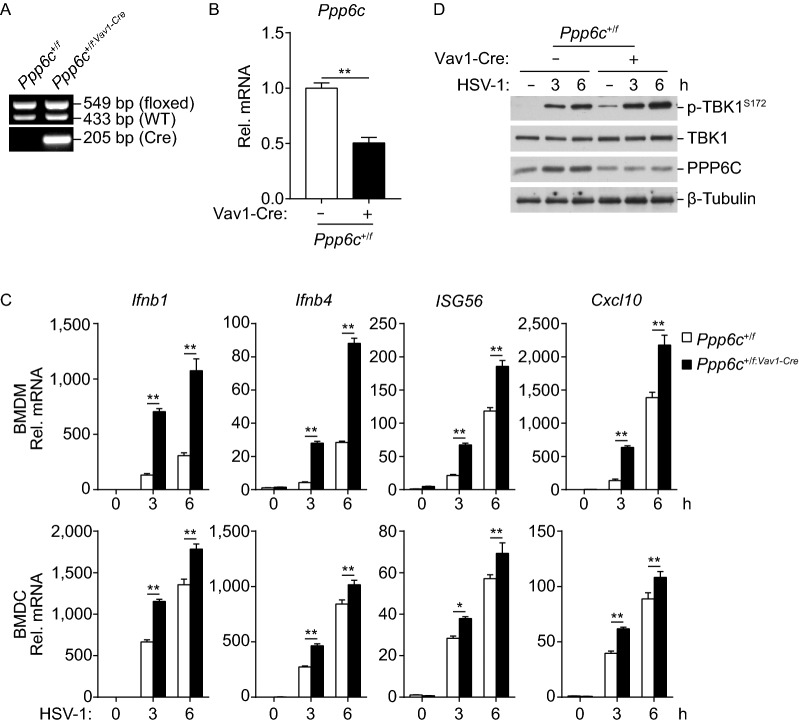
**PPP6C negatively regulates innate immune response to HSV-1 in mouse immune cells**. (A) Genotyping of *Ppp6c* conditional knockout mice by PCR. (B) mRNA levels of *Ppp6c* in wild-type and *Ppp6c*^+/*f*: *Vav1-Cre*^ BMDMs. The indicated BMDMs (1 × 10^6^) were collected for qPCR analysis. Graph shows mean ± SEM, *n* = 3. **P < 0.01. Data were analyzed using a Student’s unpaired *t*-test with Prism GRAGHPAD 7. (C) Knockdown of PPP6C increases HSV-1-induced transcription of downstream genes in mouse immune cells. The indicated BMDMs or BMDCs (1 × 10^6^) were left untreated or infected with HSV-1 (MOI = 1) for the indicated times before qPCR analysis of mRNA levels of the indicated genes. Graph shows mean ± SEM, *n* = 3. *P < 0.05, **P < 0.01. Data were analyzed using two-way ANOVA with Prism GRAGHPAD 7. (D) Knockdown of PPP6C increases HSV-1-induced phosphorylation of TBK1 in BMDMs. The indicated BMDMs (1 × 10^6^) were left untreated or infected with HSV-1 (MOI = 1) for the indicated times before immunoblot analysis with the indicated antibodies

It has been reported that PPP6C homozygous null mutations are early embryonic lethal in mice (Ogoh et al., 2016). To investigate the functions of PPP6C *in vivo*, we crossed the *Ppp6c*^*f*/*f*^ mice with *Vav1-Cre* mice to obtain *Ppp6c* hematopoietic-specific knockout strain (*Ppp6c*^*f*/*f*: *Vav1-Cre*^). Unexpectedly, of the 200 examined offspring from the *Ppp6c*^*f*/*f*^ and *Ppp6c*^+/*f: Vav1-Cre*^ parents, only 4 mice were *Ppp6c*^*f*/*f*: *Vav1-Cre*^ genotype, and these 4 mice all died during 10 to 15 days after birth. Thus, we used *Ppp6c*^+/*f*^ and *Ppp6c*^+/*f*: *Vav1-Cre*^ for further investigation. The successful targeting of *Ppp6c* gene was verified by genotyping (Fig. 2A). qPCR experiments indicated that the mRNA levels of *Ppp6c* gene in *Ppp6c*^+/*f*: *Vav1-Cre*^ bone marrow derived macrophages (BMDMs) were reduced to ~50% of the wild-type cells (Fig. 2B). We found that knockdown of PPP6C enhanced HSV-1-induced transcription of *Ifnb1*, *Ifna4*, *Isg56* and *Cxcl10* genes in BMDMs and bone marrow-derived dendritic cells (BMDCs) (Fig. 2C). Biochemical analysis showed an enhanced phosphorylation of TBK1 S172, which is a hallmark of its activation, in PPP6C-knockdown BMDMs in response to HSV-1 (Fig. 2D). These data suggest that PPP6C negatively regulates DNA virus-induced transcription of downstream antiviral genes in mouse immune cells.

### PPP6C dephosphorylates cGAS

**Figure 3:**
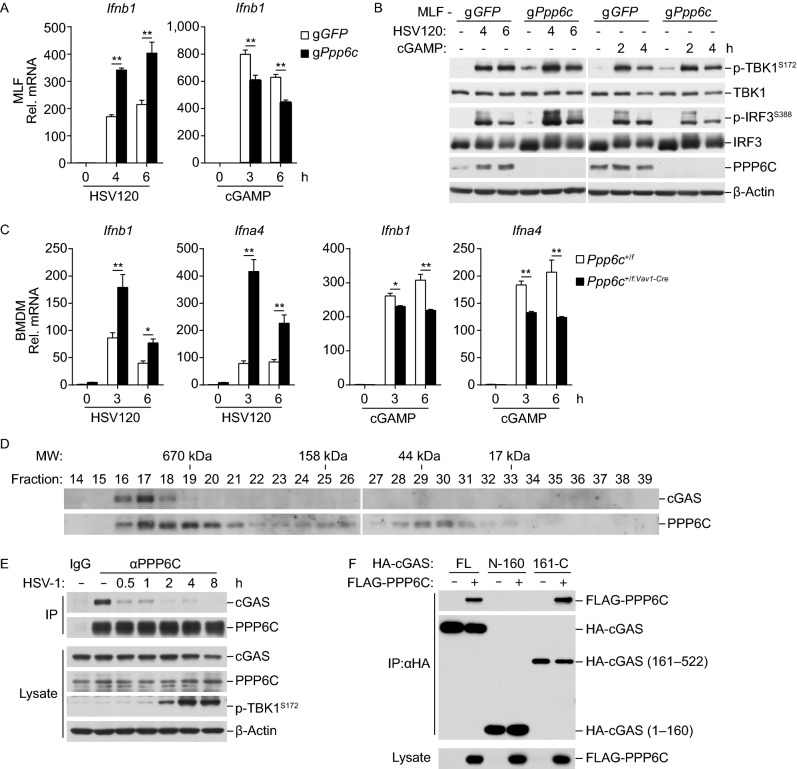
**PPP6C targets cGAS in DNA virus-induced signaling**. (A) Deficiency of PPP6C enhances HSV120- but not 2’3’-cGAMP-induced transcription of *Ifnb1* gene. The control or PPP6C-deficient MLFs (1 × 10^6^) were left untreated or treated with the indicated stimuli for the indicated times before qPCR analysis of mRNA levels of the *Ifnb1* gene. Graph shows mean ± SEM, *n* = 3. **P < 0.01. Data were analyzed using two-way ANOVA with Prism GRAGHPAD 7. (B) Deficiency of PPP6C increases HSV120- but not 2’3’-cGAMP-induced phosphorylation of TBK1 and IRF3. The control or PPP6C-deficient MLFs (1 × 10^6^) were left untreated or treated with the indicated stimuli for the indicated times before immunoblot analysis with the indicated antibodies. (C) Knockdown of PPP6C increases HSV120- but not 2’3’-cGAMP-induced transcription of *Ifnb1* and *Ifna4* gene in BMDMs. The control or PPP6C-deficient BMDMs (1 × 10^6^) were left untreated or treated with the indicated stimuli for the indicated times before qPCR analysis. Graph shows mean ± SEM, *n* = 3. *P < 0.05, **P < 0.01. Data were analyzed using two-way ANOVA with Prism GRAGHPAD 7. (D) cGAS and PPP6C exist in overlapping high molecular-weight complexes. Cytoplasmic lysate of MLFs were fractionated by gel filtration and the fractions were analyzed by immunoblots with the indicated antibodies. (E) Endogenous association of PPP6C and cGAS. MLFs (1 × 10^7^) were left untreated or infected with HSV-1 (MOI = 2) for the indicated times. Cytoplasmic lysates were subjected to co-immunoprecipitation and immunoblotting analysis with the indicated antibodies. (F) PPP6C interacts with the C-terminus of cGAS. HEK293T cells were transfected with the indicated plasmids for 16 h before co-immunoprecipitation and immunoblotting analysis with the indicated antibodies

To investigate the mechanisms on how PPP6C is involved in innate antiviral response to DNA virus, we investigated the effects of PPP6C-deficiency on transcription of downstream antiviral genes induced by transfected HSV120 and 2’3’-cGAMP. The results indicated that PPP6C-deficiency enhanced HSV120- but not 2’3’-cGAMP-triggered transcription of *Ifnb1* gene (Fig. 3A) and phosphorylation of TBK1 and IRF3 (Fig. 3B) in MLF cells. In addition, knockdown of PPP6C in BMDMs also enhanced HSV120 but not 2’3’-cGAMP-triggered transcription of *Ifnb1* and *Ifna4* genes (Fig. 3C). These data suggest that PPP6C inhibits cGAS activity. Gel-filtration experiments indicated that cGAS and PPP6C exist in overlapping high molecular-weight complexes (>670 kDa) in MLF cells (Fig. 3D). Endogenous coimmunoprecipitation experiments indicated that PPP6C was constitutively associated with cGAS in un-infected cells, but their association was dramatically decreased upon HSV-1 infection (Fig. 3E). Domain mapping experiments indicated that PPP6C was associated with the C-terminal catalytic domain (aa 161–522) of cGAS (Fig. 3F). These data suggest that PPP6C acts at the cGAS level.

**Figure 4:**
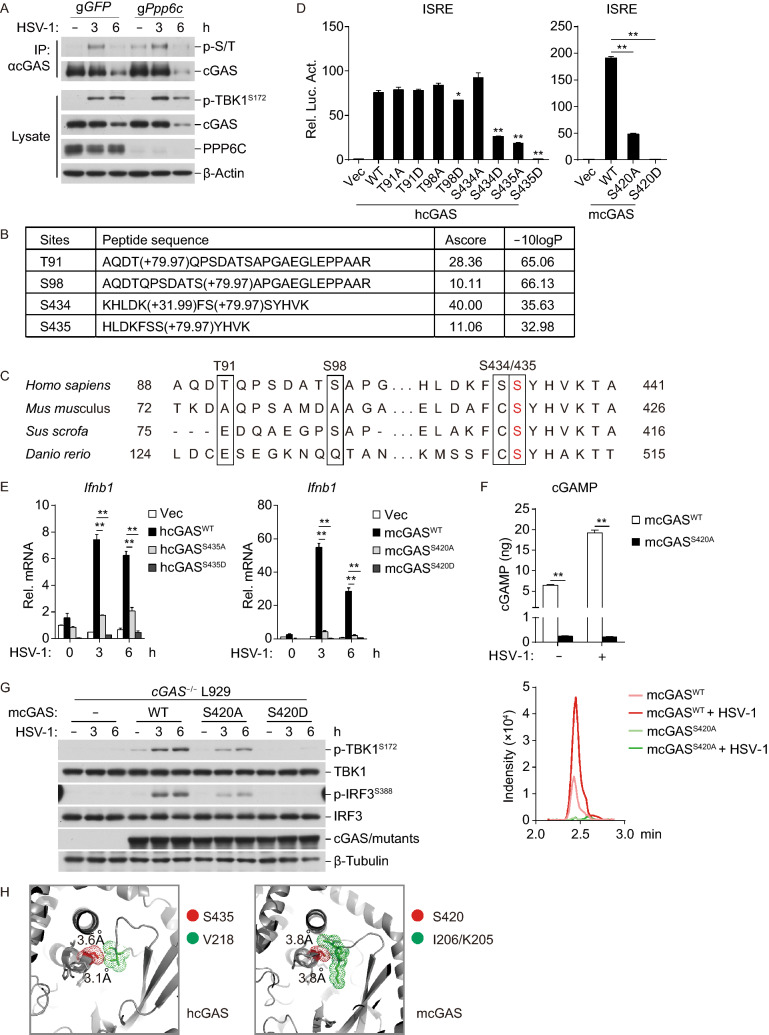
**mcGAS S420 or hcGAS S435 phosphorylation is essential for cGAS activity**. (A) Serine/threonine phosphorylation of cGAS is increased in PPP6C-deficient MLFs. The control or PPP6C-deficient MLFs (1 × 10^7^) were left untreated or infected with HSV-1 (MOI = 2) for the indicated times. Cytoplasmic lysates were subjected to co-immunoprecipitation and immunoblotting analysis with the indicated antibodies. (B) Phosphorylated peptides of hcGAS derived from okadaic acid-treated cells. HEK293T cells (1 × 10^8^) transfected with FLAG-tagged cGAS and PPP6C or transfected with FLAG-tagged cGAS treated with OA (100 nmol/L) were harvested. Cell lysates were immunoprecipitated with anti-FLAG coupled beads. Phosphorylated peptides of the immunoprecipitates were analyzed by mass spectrometry. The list shows the phosphorylated peptides of hcGAS found in OA-treated but not the other non-treated samples. (C) Alignments of potential serine/threonine phosphorylation residues of cGAS. The conserved serine residue is highlighted as red. (D) Mutation of hcGAS S435 or mcGAS S420 impairs its activity. HEK293T cells were transfected with ISRE reporter plasmid, and expression plasmids for MITA and cGAS or its mutants for 16 h before luciferase assays being performed. Graph shows mean ± SEM, *n* = 3. *P < 0.05, **P < 0.01. Data were analyzed using a Student’s unpaired *t*-test with Prism GRAGHPAD 7. (E) Effects of the serine mutation of cGAS on HSV-1-triggered transcription of *Ifnb1* gene. cGAS-deficient L929 cells were reconstituted with hcGAS (S435A/D), mcGAS (S420A/D), or their wild-type counterparts. The cells were then infected with HSV-1 (MOI = 1) for the indicated times before qPCR analysis of mRNA levels of the *Ifnb1* gene. Graph shows mean ± SEM, *n* = 3. **P < 0.01. Data were analyzed using two-way ANOVA with Prism GRAGHPAD 7. (F) Effects of mcGAS S420 mutation on HSV-1-triggered synthesis of cGAMP. cGAS-deficient L929 cells were reconstituted with mcGAS (S420A/D) or its wild-type counterpart. The cells were then infected with HSV-1 (MOI = 1) for 3 h before being collected for cGAMP quantification by LC. Graph shows mean ± SEM, *n* = 3. **P < 0.01. Data were analyzed using two-way ANOVA with Prism GRAGHPAD 7. (G) Effects of mcGAS S420 mutation on HSV-1-induced phosphorylation of MITA, TBK1 and IRF3. cGAS-deficient L929 cells were reconstituted with mcGAS (S420A/D) or its wild-type counterpart. The cells were then infected with HSV-1 (MOI = 1) for the indicated times before immunoblot analysis with the indicated antibodies. (H) Conformational structures around the conserved serine residue of hcGAS (PDB entry 4LEV) and mcGAS (PDB entry 4K9B)

We next examined whether PPP6C dephosphorylates cGAS. Coimmunoprecipitation and immunoblotting analysis indicated that serine/threonine phosphorylation of cGAS was undetectable in wild-type MLFs but was detected in PPP6C-deficient MLFs (Fig. 4A). HSV-1 infection induced serine/threonine phosphorylation of cGAS to comparable levels in wild-type and PPP6C-deficient MLFs (Fig. 4A). These results suggest that PPP6C dephosphorylates cGAS in un-infected cells and dissociates with cGAS after viral infection.

To identify the potential residues of cGAS that are dephosphorylated by PPP6C, we analyzed phosphorylation sites of human cGAS (hcGAS) immunoprecipitated from okadaic acid-treated HEK293T cells. These experiments indicated that four residues in hcGAS, including T91, S98, S434 and S435 were phosphorylated in okadaic acid-treated but not in un-treated HEK293T cells (Fig. 4B). Among the four residues, only S435 of hcGAS, which is corresponding to S420 in murine cGAS (mcGAS) is conserved across species (Fig. 4C). To investigate the functions of phosphorylation of the four residues, we made hcGAS mutants in which the four S/T residues are individually mutated to alanine (A), which simulates the un-phosphorylated state, or aspartic acid (D), which mimics the phosphorylated state. Reporter assays indicated that mutation of S435 but not T91, S98 or S434 of hcGAS to alanine markedly inhibited its ability to activate ISRE (Fig. 4D). Unexpectedly, mutation of S435 of hcGAS to aspartic acid completely impaired its ability to activate ISRE (Fig. 4D). Consistently, mutation of S420 of mcGAS to alanine or aspartic acid markedly reduced or completely impaired its ability to activate ISRE (Fig. 4D). To further investigate the functions of cGAS phosphorylation, we reconstituted wild-type human and murine cGAS and their mutants to cGAS-deficient mouse fibroblast L929 cells via a pseudotyped retroviral-mediated gene transfer approach. qPCR experiments indicated that reconstitution with wild-type but not the hcGAS S435 or mcGAS S420 mutants restored transcriptional induction of *Ifnb1* gene after HSV-1 infection (Fig. 4E). In addition, HSV-1-induced cGAMP production (Fig. 4F), and phosphorylation of TBK1 S172 and IRF3 S388 (which are hallmarks of their activation) (Fig. 4G), were markedly reduced or completely impaired in cells reconstituted with mcGAS S420 mutants in comparison with those reconstituted with wild-type mcGAS. These results suggest that phosphorylation of hcGAS S435 or mcGAS 420 is critical for their abilities to mediate innate antiviral responses.

Since mutation of serine in a phosphoprotein to alanine simulates its un-phosphorylated state, while mutation of serine to aspartic acid usually mimics its phosphorylated state. It is unexpected that mutation of hcGAS S435 or mcGAS S420 to aspartic acid had even more dramatic negative effects on its activity than its mutation to alanine. Structural analysis indicated there is limited space around hcGAS S435 or mcGAS S420. The distance of this serine to nearby residues is ~3.6 Å, suggesting that there might be hydrogen bonds between the serine and nearby residues (Fig. 4H) (Gao et al., 2013; Li et al., 2013). It is possible that mutation of hcGAS S435 or mcGAS S420 to aspartic acid, which has a long side chain, may cause a disruptive conformational change of cGAS that leads to its inactivation.

**Figure 5:**
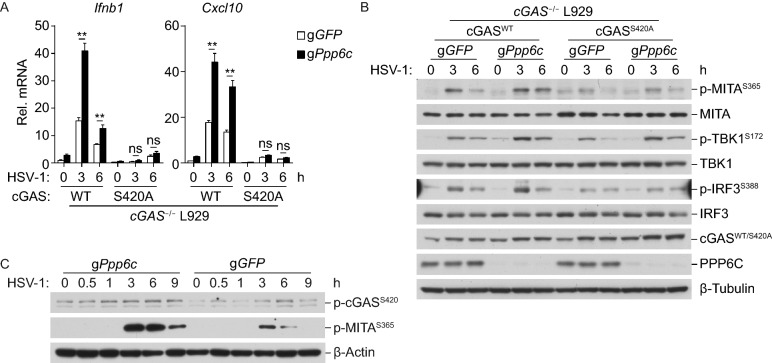
**PPP6C dephosphorylates mcGAS at S420**. (A) Effects of mcGAS S420 mutation on HSV-1-induced transcription of downstream genes. cGAS-deficient L929 cells were reconstituted with mcGAS (S420A) or its wild-type counterpart. The cells (1 × 10^6^) were left untreated or infected with HSV-1 (MOI = 1) for the indicated times before qPCR analysis of mRNA levels of the indicated genes. Graph shows mean ± SEM, *n* = 3. **P < 0.01; ns, not significant. Data were analyzed using two-way ANOVA with Prism GRAGHPAD 7. (B) Effects of PPP6C-deficiency on phosphorylation of MITA, TBK1 and IRF3 mediated by wild-type mcGAS and mcGAS (S420A). PPP6C was knockout by CRISPR-Cas9 system in wild-type mcGAS- or mcGAS(S420A)-reconstituted L929 cells. The cells (1 × 10^6^) were left untreated or infected with HSV-1 (MOI = 1) for the indicated times before immunoblotting analysis with the indicated antibodies. (C) Deficiency of PPP6C enhances HSV-1-induced phosphorylation of endogenous mcGAS at S420 in MLFs. PPP6C was knockout by CRISPR-Cas9 system in MLFs. The cells (1 × 10^6^) were left untreated or infected with HSV-1 (MOI = 1) for the indicated times before immunoblotting analysis with the indicated antibodies

To verify that PPP6C indeed targets mcGAS S420 for dephosphorylation, we further knocked out PPP6C in wild-type mcGAS or mcGAS(S420A)-reconstituted cGAS-deficient L929 cells. qPCR experiments indicated that PPP6C-deficiency significantly enhanced transcription of *Ifnb1* and *Cxcl10* genes in wild-type mcGAS- but not mcGAS (S420A)-reconstituted cells (Fig. 5A). Immunoblot analysis indicated that PPP6C-deficiency increased HSV-1-induced phosphorylation of MITA S365, TBK1 S172 and IRF3 S388 in wild-type cGAS- but not mcGAS (S420A)-reconstituted L929 cells (Fig. 5B). Furthermore, endogenous cGAS was phosphorylated at S420 after HSV-1 infection, and PPP6C-deficiency increased HSV-1-induced phosphorylation of cGAS at S420, as well as phosphorylation of MITA at S365 (Fig. 5C). These data further confirm that PPP6C targets mcGAS S420 for dephosphorylation.

### Phosphorylation of mcGAS at S420 is essential for its binding to GTP

**Figure 6:**
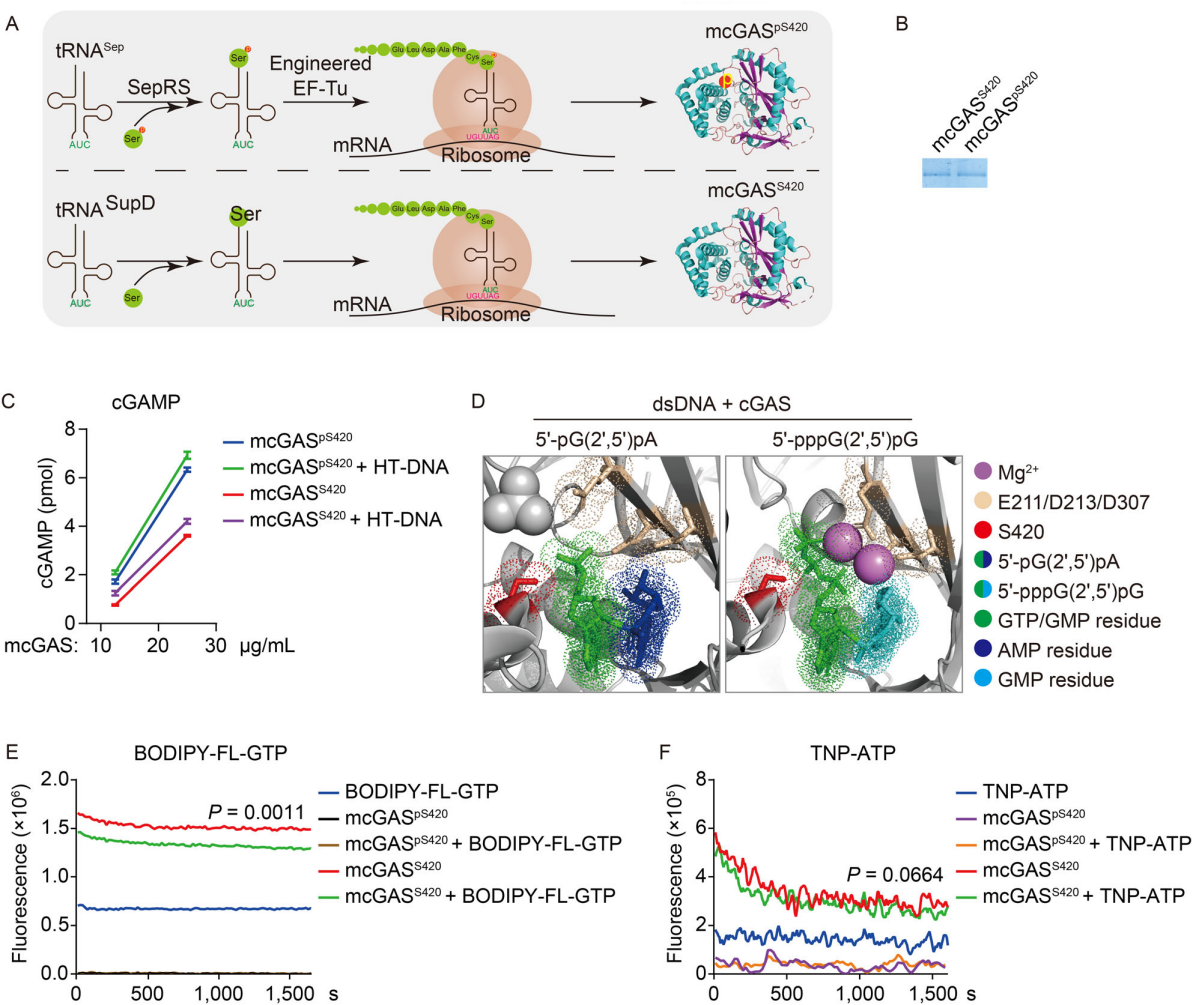
**Phosphorylation of mcGAS at S420 is essential for its binding to GTP**. (A) A schematic description for generation of recombinant S420 phosphorylated mcGAS protein or wild-type cGAS in an engineered *E*. *coli* BL21 strain with a Sep-accepting tRNA (tRNASep), its cognate phosphoseryl-tRNA synthase (SepRS), elongation factor-Tu (EF-Sep) and supD. (B) Purification of mcGAS^S420^ and mcGAS^pS420^ protein. The purified proteins were visualized by Colloidal Blue Staining (Invitrogen^TM^). (C) cGAS^pS420^ has higher enzymatic activity than cGAS^S420^. The recombinant proteins were mixed with HT-DNA, ATP and GTP. Synthesized cGAMP was detected by malachite green phosphate detection kit (Cell Signaling Technology). Graph shows mean ± SEM, *n* = 3. (D) Conformational structure of mcGAS with 5’-pG(2’,5’)pA and 5’-pppG(2’,5’)pG(PDB entry 4K9B). (E) cGAS^pS420^ has higher binding affinity to GTP than cGAS^S420^. The recombinant cGAS^S420^ or cGAS^pS420^ were mixed with HT-DNA and BODIPY-FL-GTP and then the fluorescence intensities of EX485 nm/EM520 nm were records immediately ~100 cycles every 16 s for about 30 min. Data shown are average of three technical repeats. (F) cGAS^pS420^ and cGAS^S420^ have comparable affinity to ATP. The recombinant cGAS^S420^ or cGAS^pS420^ were mixed with HT-DNA and TNP-ATP and then The fluorescence intensities of EX410 nm/EM561 nm were records immediately ~100 cycles every 16 s for about 30 min. Data shown are average of three technical repeats

To unambiguously determine the effects of phosphorylation on mcGAS S420, we utilized an *E*. *Coli* based system to genetically incorporate phosphoserine (Sep) into recombinant mcGAS protein at S420 to create mcGAS^pS420^ (Pirman et al., 2015). Sep is not genetically encoded in eukaryotes. However, the *E*. *coli* strain C321.ΔA engineered with the ΔmutS:zeo, ΔtolC, Δbla:tolC, SerB-/ΔSerB genotypes harbors a Sep-accepting tRNA (tRNASep), its cognate phosphoseryl-tRNA synthase (SepRS) which charges Sep onto a UAG-decoding tRNA^Sep^, and an engineered EF-Tu (EF-Sep) that delivers Sep-tRNA^Sep^ to the ribosomes. The *E*. *coli* strain was transformed with a bacterial expression vector for SBP-tagged mcGAS^pS420^ (mcGAS cDNA with an UAG codon for S420). Similarly, we employed a known amber suppressor tRNA SupD to incorporate serine at the UAG codon to obtain wild-type mcGAS^S420^ (Fig. 6A). We purified the recombinant mcGAS^S420^ and mcGAS^pS420^ from *E*. *coli* (Fig. 6B) and measured their nucleotidyltransferase activity by a pyrophosphatase-malachite green-coupled assay (Hooy and Sohn, 2019). In these *in vitro* experiments, mcGAS^pS420^ exhibited a much higher enzymatic activity to synthesize cGAMP than mcGAS^S420^ either with or without addition of DNA to the reaction (Fig. 6C). In these experiments, we noticed that the enzymatic activity of mcGAS^pS420^ was only slightly increased by addition of DNA to the reaction (Fig. 6C). The simplest explanation for this observation is that mcGAS^pS420^ has already bound to bacterial DNA during the preparation of the recombinant protein. These results suggest that phosphorylation of mcGAS at S420 is important for its enzymatic activity.

Because mcGAS S420 is located in its catalytic pocket, where cGAS utilizes ATP and GTP as substrates to synthesize 2’3’-cGAMP (Fig. 6D) (Gao et al., 2013), we examined whether mcGAS S420 phosphorylation affects its binding to ATP and GTP. To do this, we utilized BODIPY-FL-GTP and TNP-ATP, which are nucleotide analog carrying fluorophore BODIPY-FL and TNP respectively. The fluorescence quantum yield of BODIPY-FL-GTP is enhanced upon binding to GTP-binding proteins (Willard et al., 2005). TNP-ATP is only fluorescent when bound to the nucleotide-binding site of proteins (Hiratsuka, 1975). We mixed cGAS^S420^ or cGAS^pS420^ with BODIPY-FL-GTP or TNP-ATP and then monitored the fluorescent quantum yields. The results indicated that cGAS^pS420^ bound to BODIPY-FL-GTP with a significantly higher affinity than cGAS^S420^ (Fig. 6E). In similar experiments, cGAS^S420^ and cGAS^pS420^ showed comparable affinities to bind TNP-ATP (Fig. 6F). It has been reported that cGAS contains a single active site for stepwise formation of GpA and ApG linkages. Structural analysis of the ternary complex of dsDNA/cGAS/5’-pG(2’,5’)pA, which mimics the intermediate product of first stepwise formation of GpA linkage using GMP instead of GTP for the crystallizing, indicates that S420 is important for supporting the GMP residue (green) of the intermediate product while E211/D213/D307 or cations are important for supporting the ATP residue (blue) (Fig. 6D, left panel). Consistently, in the ternary complex of dsDNA/cGAS/5’pppGpG, which contains the phosphodiester bond formed 5’-pppGpG and represents the similar structure with the intermediate product, the γ-phosphate of GTP residue is hydrogen bonded to polar chains of S420 (Fig. 6D, right panel) (Gao et al., 2013). These results suggest that phosphorylation of mcGAS S420 enhanced its GTP binding ability, which is consistent with previous structural data.

## Discussion

By monitoring microbial DNA, mis-located mtDNA or nuclear DNA in the cytosol, cGAS plays a crucial role in host defense to microbial pathogens as well as abnormal/damaged cells. In this study, we demonstrate that dephosphorylation of a catalytic serine residue of cGAS by the phosphatase PPP6C impairs its substrate binding and enzymatic activity, therefore provides a mechanism for keeping the DNA sensor cGAS inactive in the absence of infection to avoid autoimmune response.

PPP6C was identified as a negative regulator of DNA virus-triggered transcription of *Ifnb1* gene by functional screens with the CRISPR-Cas9 system. Deficiency of PPP6C increased induction of downstream antiviral genes triggered by HSV-1 infection and transfected DNA but not cGAMP. Endogenous co-immunoprecipitation experiments indicated that PPP6C was constitutively associated with cGAS and the association was rapidly decreased upon HSV-1 infection. In addition, phosphorylation of cGAS in PPP6C-deficient cells was markedly enhanced compared to the control cells. These results suggest that PPP6C constitutively associates with cGAS to suppress its phosphorylation and activity in un-infected cells, and they rapidly dissociate with cGAS upon DNA virus infection to ensure immediate activation of innate antiviral response.

Our experiments suggest that PPP6C targets hcGAS S435 (mcGAS S420) for dephosphorylation. Deficiency of PPP6C potentiates HSV-1 induced transcription of *Ifnb1* gene and phosphorylation of MITA, TBK1 and IRF3 in wild-type mcGAS- but not mcGAS(S420A)-reconstituted L929 cells. Mutations of mcGAS S420 to alanine markedly reduced its ability to mediate innate antiviral response. *In vitro* experiments suggest that recombinant S420-phosphorylated mcGAS exhibits a significantly higher affinity to GTP than its un-phosphorylated counterpart. In similar experiments, mcGAS S420 phosphorylation has no effects on its binding affinity to ATP. Structural analysis suggests that S420 is close to the GTP binding space but far away from the ATP binding space in the catalytic pocket of mcGAS. These results suggest that phosphorylation of S420 in the catalytic pocket of mcGAS is specifically required for its binding to GTP but not ATP.

cGAS acts as a universal cytosolic DNA sensor which leads to activation of innate antiviral immune responses. It has been demonstrated that some severe autoimmune diseases are related to mis-activation of cGAS. Therefore, the activity of cGAS is tightly and spatiotemporally regulated to balance host immune homeostasis. Previous studies on cGAS regulation have focused on the mechanisms of its DNA binding, formation of cGAS liquid droplets or its stability. We demonstrate a serine residue phosphorylation of cGAS regulates its GTP substrate binding affinity, which represents a new type of regulation of cGAS activity. Our findings suggest that in un-infected cells, PPP6C is constitutively associated with cGAS and mediates its dephosphorylation of a catalytic pocket serine. This impairs the ability of cGAS to bind to its substrate GTP, leading to its inactivation. Upon DNA virus infection, PPP6C is promptly disassociated with cGAS, which conditions for cGAS phosphorylation at the serine residue in its catalytic pocket and promotes its substrate binding and enzymatic activity, leading to cGAMP-MITA-mediated innate immune response. Further investigations are needed to decipher the molecular events leading to phosphorylation of the catalytic pocket serine residue and enzymatic activation of cGAS upon DNA virus infection.

Recent studies have pointed to a role of cGAS in tumorigenesis. It has been demonstrated that nuclear cGAS is recruited to double-stranded breaks and interacts with PARP1 via poly(ADP-ribose), leading to suppression of homologous recombination and thereby promotion of tumor growth (Liu et al., 2018). Interestingly, PPP6C has been reported to be involved in both homology-directed repair of DNA double-strand breaks and tumor developments. It would be interesting to investigate where PPP6C regulates cGAS-mediated tumorigenesis in future studies.

## Materials and Methods

### Reagents, antibodies, cells and viruses

Phusion^®^ High-Fidelity DNA Polymerase (NEW ENGLAND BioLabs); GM-CSF (PeproTech); and 2’3’-cGAMP (InvivoGen); digitonin and DNase I (Sigma-Aldrich); Lipofectamine 2000, M-MLV Reverse Transcriptase, BODIPY-FL-GTP, TNP-ATP and Colloidal Blue Staining Kit (Invitrogen); RiboLock RNase Inhibitor, Pyrophosphatase, NE-PER™ Nuclear and Cytoplasmic Extraction Reagents (Thermo Scientific); protease inhibitor cocktail (Roche); polybrene (Millipore); RNAiso Plus (TaKaRa); SYBR Green mix (Bio-Rad); Malachite Green Phosphate Detection Kit (Cell Signaling Technology); Okadaic Acid (Sigma); Dual-Specific Luciferase Assay Kit (Promega); ELISA kits for human IFN- (PBL); mouse antibodies against HA (Origene), FLAG and -Actin (Sigma-Aldrich), -Tubulin (Life Technologies); rabbit antibodies against phospho-MITA (S366), MITA, phospho-IRF3 (S396), cGAS (Cell Signaling Technology), phospho-TBK1 (S172), TBK1, PPP6C and phospho-S/T (Abcam) (Proteintech) were purchased from the indicated manufacturers. Rabbit antisera against murine IRF3 were raised using the full-length recombinant protein as an antigen. HEK293T cells were originally provided by Dr. Gary Johnson (National Jewish Health, Denver, CO). MLFs were obtained from C57BL/6 mice and immortalized by SV40. THP-1 cells were obtained from the American Type Culture Collection. cGAS-deficient L929 cells were provided by Dr. Jiahuai Han (Xiamen University). SeV (Cantell strain, Charles River Laboratories) and HSV-1 (KOS strain, China Center for Type Culture Collection) were obtained from the indicated resources. SV40 virus was provided by Dr. Zhiying Song (Wuhan University).

### Constructs

Expression plasmids for FLAG-tagged PPP6C and PPP6C (H114A) were constructed with the pLOV-CMV-eGFP-2A-EF1a-PuroR vector. Expression plasmids for cGAS and its mutants were constructed with the pMSCV-PuroR vector. Guide-RNA (gRNA) plasmids targeting the PPPs were constructed with the lentiCRISPR V2 vector (provided by Dr. Shuwen Wu (Wuhan University)). SBP-tagged mcGAS S420TAG was constructed with the GFP E17TAG vector (which was a gift from Jesse Rinehart (Addgene plasmid #68295)). SepOTSκ and supD was a gift from Jesse Rinehart (Addgene plasmid #68291 and #68307).

### *Ppp6c* conditional knockout mice and genotyping

*Ppp6c* conditional knockout mice were a gift from Dr. Qingyuan Sun. Genotyping by PCR was performed using the following primers: P8: 5′-GAGGGCAGAGGATGGGGTCACA-3′; P3: 5′-ATCTCTGAACCAATTCTGGAG-3′.

Amplification of the WT allele with primers P8 and P3 generates a 433-bp fragment, whereas amplification of the disrupted allele with primers P8 and P3 generates a 549-bp fragment. To generate *Ppp6c* hematopoietic-specific knockout mice, *Ppp6c*^+/−^ mice were bred to *Vav1-Cre* mice to generate *Ppp6c*^*f*/*f*: *Vav1-Cre*^ mice. Genotyping of the *Vav1-Cre* mice by PCR was performed using the following primers: Cre (205 bp): Foward-CGTATAGCCGAAATTGCCAG; Reverse-CAAAACAGGTAGTTATTCGG.

All animal experiments were performed in accordance with the Wuhan University Animal Care and Use Committee guidelines.

### Preparation of BMDMs and BMDCs

Monocytes were isolated from mouse tibia and femur. For preparation of BMDMs, the monocytes were cultured in 10% M-CSF-containing conditional medium from L929 cells for 3 days. For preparation of BMDCs, the monocytes were cultured in medium containing murine GM-CSF (50 ng/mL) for 7 days.

### DNA oligonucleotides

The following oligonucleotides were used to stimulate cells. HSV120: 5′-AGACGGTATATTTTTGCGTTATCACTGTCCCGGATTGGACACGGTCTTGTGGGATAGGCATGCCCAGAAGGCATATTGGGTTAACCCCTTTTTATTTGTGGCGGGTTTTTTGGAGGACTT-3′. The following oligonucleotides were used to construct the respective gRNA plasmids. g*Ppp1ca*: #1-5′-CTGACAGAGAACGAGATCCG-3′, #2-5′-AGCAGATTAGGCGTATTATG-3′, #3-5′-CATACTCAAACAGCCGTAGA-3′; g*Ppp1cb*: #1-5′-CCAGTACGAGGATGTCGTCC-3′, #2-5′-ACCTGTATCAGGTACGTCAG-3′, #3-5′-CCCACTGACGTACCTGATAC-3′; g*Ppp1cc*: #1-5′-CACTCACCACATATCTTGAG-3′, #2-5′-TCACCCTTAGGTGACATCCA-3′, #3-5′-ACAGTGAGAGGGTCCAAGCC-3′; g*Ppp2ca*: #1-5′-CCGAGCACTCGATCGCCTAC-3′, #2-5′-ACATCGAACCTCTTGAACGT-3′, #3-5′-GGGATATCTCCTCGGGGAGC-3′; g*Ppp2cb*: #1-5′-GAGCGCATCACAATATTGCG-3′, #2-5′-CGCAATATTGTGATGCGCTC-3′, #3-5′-GAACTTCTTGCAAGCGATCC-3′; g*Ppp3ca*: #1-5′-GCTGGTTCATTAGCGCAGCC-3′, #2-5′-GCAGTCGAAGGCATCCATAC-3′, #3-5′-CCGACAGGAAAAAAACTTGC-3′; g*Ppp3cb*: #1-5′-TCAGATGTCAGCCGATGAGT-3′, #2-5′-GAAGTAGAAGCTCCAATTAC-3′, #3-5′-AAGAGTCTATGAAGCTTGTA-3′; g*Ppp3cc*: #1-5′-CTTCCTTCAAAGTTAGCCGT-3′, #2-5′-TAATACTCGCTACCTCTTCC-3′, #3-5′-CGGAGCCTCCACCTCTATCA-3′; g*Ppp4c*: #1-5′-TGAGAGTCGCCAGATTACCC-3′, #2-5′-GGCGACTCTCATGATTGCCC-3′, #3-5′-TCCTCACCTTAAGAGCCAGC-3′; g*Ppp5c*: #1-5′-GATCGCGTTCTCGTAGTCCT-3′, #2-5′-TCACCGTCTCGTAGTCACGC-3′, #3-5′-TCTGTGCTCGTCACCCGCAA-3′; g*Ppp6c*: #1-5′-TCAACGCCAGTAACAGTGTG-3′, #2-5′-GATGAGTGCCAAACCAAATA-3′, #3-5′-ATGTCACCACACACTGTTAC-3′; g*Ppef1*: #1-5′-TCAGTCGAGCTCTGTACCGC-3′, #2-5′-CCGCGTTAGTCATCCAAAAT-3′, #3-5′-TCATGCTCACTACGTCTTAG-3′; g*Ppef2*: #1-5′-GATTCAAGACGTATCGAGCA-3′, #2-5′-CACCGAAGAGAGATTCGCCC-3′, #3-5′-TGTAGCGCCGGTACCATCTC-3′.

### qPCR

Total RNA was isolated for qPCR analysis to measure mRNA levels of the indicated genes according to the manufacturer’s protocol (TaKaRa). Data shown are the relative abundance of the indicated mRNA normalized to that of *ACTB*. Gene-specific primer sequences were as following. Murine *Ifnb1*, 5′-TCCTGCTGTGCTTCTCCACCACA-3′ (forward) and 5′-AAGTCCGCCCTGTAGGTGAGGTT-3′ (reverse); murine *Ifna4*, 5′-CCTGTGTGATGCAGGAACC-3′ (forward) and 5′-TCACCTCCCAGGCACAGA-3′ (reverse); murine *Cxcl10*, 5′-ATCATCCCTGCGAGCCTATCCT-3′ (forward) and 5′-GACCTTTTTTGGCTAAACGCTTTC-3′ (reverse); murine *Isg56*, 5′-ACAGCAACCATGGGAGAGAATGCTG-3′ (forward) and 5′-ACGTAGGCCAGGAGGTTGTGCAT-3′ (reverse); murine *Actb*, 5′-CATTGCTGACAGGATGCAGAAGG-3′ (forward) and 5′-TGCTGGAAGGTGGACAGTGAGG-3′ (reverse); murine *Ppp6c*, 5′-TGTGATCTGCTCTTGGAAGAGTC-3′ (forward) and 5′-TGTCAGGAACCTGACCTCCAGT-3′ (reverse); human *IFNB1*, 5′-CTTGGATTCCTACAAAGAAGCAGC-3′ (forward) and 5′-TCCTCCTTCTGGAACTGCTGCA-3′ (reverse); human *ISG56*, 5′-GCCTTGCTGAAGTGTGGAGGAA-3′ (forward) and 5′-ATCCAGGCGATAGGCAGAGATC-3′ (reverse); human *CXCL10*, 5′-GGTGAGAAGAGATGTCTGAATCC-3′ (forward) and 5′-GTCCATCCTTGGAAGCACTGCA-3′ (reverse); *STAT1*, 5′-ATGGCAGTCTGGCGGCTGAATT-3′ (forward) and 5′-CCAAACCAGGCTGGCACAATTG-3′ (reverse); and *ACTB*, 5′-CACCATTGGCAATGAGCGGTTC-3′ (forward) and 5′-AGGTCTTTGCGGATGTCCACGT-3′ (reverse).

### Transfection

HEK293T cells were transfected by standard calcium phosphate precipitation method. BMDMs, BMDCs and L929 cells were transfected by lipofectamine 2000. Briefly, BMDMs, BMDCs and L929 cells (5 × 10^5^) were seeded on 12-well plates and transfected the following day with the indicated nucleic acids (2 µg/mL) by lipofectamine 2000 according to the manufacturer’s instructions.

### Dual specific luciferase assay

Luciferase assays were performed using a Dual-Specific Luciferase Assay Kit (Promega). To normalize for transfection efficiency, pRL-TK (Renilla luciferase) reporter plasmid (0.01 µg) was added to each transfection. Firefly luciferase activities were normalized based on Renilla luciferase activities.

### Gel filtration

Cytoplasmic fractions were prepared with NE-PER^TM^ nuclear and cytosolic reagents (Thermo Scientific) with protease inhibitor cocktail procedures recommended by the manufacturer. The cytoplasmic fractions were centrifuged at 16,000 ×*g* for 10 min at 4 °C for gel filtration. Protein samples were loaded onto a size-exclusion column (Superose 6 Increase 10/300 GL) with the capacity to separate the large protein complexes. Samples were fractionated in PBS at a flow rate of 0.35 mL per min and collected as 0.5 mL fractions. Protein fractions were separated by SDS-PAGE and detected with the indicated antibodies.

### Coimmunoprecipitation and immunoblotting analysis

Cells were lysed in Triton X-100 lysis buffer (20 mmol/L Tris-HCl, pH 7.4, 150 mmol/L NaCl, 1 mmol/L EDTA, 1% Triton X-100, 10 µg/mL aprotinin, 10 µg/mL leupeptin, 1 mmol/L phenylmethylsulphonyl fluoride, PMSF). Coimmunoprecipitation and immunoblotting analysis were performed as previously described (Yan et al., 2017).

### Plaque assay

MLFs were infected with HSV-1 (MOI = 0.01) for 36 h, then collected in medium and broken by freeze-thaw cycle for 3 times. The cellular suspensions were centrifuged at 10,000 ×*g* for 5 min and the supernatants were used for plaque assays on monolayers of Vero cells seeded in 24-well plates. The cells were infected by incubation for 1 h at 37 °C with serial dilutions of the viral-containing supernatants. After 1-hour infection, 2% methylcellulose was overlaid, and the plates were incubated for about 48 h. The overlay was removed, and cells were fixed with 4% paraformaldehyde for 15 min and stained with 1% crystal violet for 30 min before plaque counting.

### ELISA

The culture media or the sera were collected for measurement of IFN-β cytokines by ELISA according to the manufacturer’s protocol.

### cGAMP extraction and quantification

The indicated L929 cells (5 × 10^7^) were left untreated or infected with HSV-1 (MOI = 2) for 3 h. Cells were then homogenized by dunce homogenizer in hypotonic buffer (10 mmol/L Tris-HCl, pH 7.4, 10 mmol/L KCl, 1.5 mmol/L MgCl_2_). After centrifugation at 13,000 rpm for 20 min, the supernatant was heated at 95 °C for 10 min and centrifuged at 13,000 rpm for another 10 min to remove denatured proteins. The heat-resistant supernatants containing cGAMP were delivered for cGAMP measurements. In brief, cGAMP analysis was performed on an Ultimate 3000 UHPLC Dionex (Sunnyvale, CA) coupled with a TSQ Quantiva, Thermo Fisher (Waltham, MA). The chromatography separation was performed on a Waters C18 column (100 × 2.1 mm i.d., 1.8 µm) at 40 °C. Selective reaction monitoring (SRM) and the appropriate product ions were chosen to quantify cGAMP. The optimal conditions for ionization source were as following: positive ion: 3,500 V, ion transfer tube temperature 350 °C, vaporizer temperature 300 °C, aux gas 10 Arb, sheath gas 20 Arb, sweep gas 4 Arb.

### *In vitro* cGAMP synthesis and quantification

The protocols for *in vitro* cGAMP synthesis and quantification were previously described (Sun et al., 2013; Hooy and Sohn, 2019). To measure cGAS activity, recombinant cGAS^S420^ or cGAS^pS420^ were mixed with buffer A (20 mmol/L Hepes, pH 7.2, 5 mmol/L MgCl_2_, 50 µmol/L ATP, 50 µmol/L GTP, 0.1 mmol/L EGTA and 0.25 U/mL Pyrophosphatase) in the presence or absence of 1 µg/mL HT-DNA. After incubation at 37 °C for 1 h, the free inorganic phosphate hydrolyzed by pyrophosphatase from pyrophosphate, which is generated during the synthesis of cGAMP, then forms a green molybdophosphoric acid complex with malachite green. Formation of this complex is monitored and quantified by measuring the absorbance at 640 nm.

### GTP and ATP binding assay

The recombinant cGAS^S420^ or cGAS^pS420^ protein was mixed with 1 µg/mL HT-DNA, 10 nmol/L BODIPY-FL-GTP or 10 µmol/L TNP-ATP in assay reaction buffer (10 mmol/L Tris-HCl, pH 8.0, 10 mmol/L MgCl_2_). The fluorescence intensities (EX485 nm/EM520 nm for BODIPY-FL-GTP and EX410 nm/EM561 nm for TNP-ATP) were records immediately ~100 cycles every 16 s for about 30 min.

### Digitonin permeabilization

The cells were treated with 2’3’-cGAMP in digitonin permeabilization solution (50 mmol/L HEPES pH 7.0, 100 mmol/L KCl, 3 mmol/L MgCl_2_, 0.1 mmol/L DTT, 85 mmol/L Sucrose, 0.2% BSA, 1 mmol/L ATP, 0.1 mmol/L GTP and 10 µg/mL digitonin) at 37 °C for 30 min. The cells were then incubated in regular medium for the indicated times before qPCR or immunoblotting analysis.

### Lentiviral-mediated gene transfer

Reconstitution of PPP6C or cGAS and their mutants into MLFs or L929 cells was performed by lentiviral-mediated gene transfer. Briefly, HEK293T cells plated on 100-mm dishes were transfected with the pLOV-CMV-eGFP-2A-EF1a-PuroR expression plasmids (8 µg) together with the pVSV-G (4 µg), pRSV-REV (4 µg) and pMDL g/p RRE (4 µg) plasmids. Two days after transfection, the recombinant viruses were harvested and used to infect the indicated cells in the presence of polybrene (8 µg/mL). Another 12 h later, the infected cells were selected by puromycin (2 µg/mL for MLFs and THP-1 cells, and 8 µg/mL for L929 cells).

### CRISPR-Cas9 knockout

Double-stranded oligonucleotides corresponding to the target sequences were cloned into the lentiCRISPR V2 plasmid, which was transfected with packaging plasmids into HEK293T cells. Two days after transfection, the viruses were harvested and used to infect THP1, MLF and L929 cells. The infected cells were selected with puromycin (2 µg/mL for MLFs and THP-1 cells, and 8 µg/mL for L929 cells) for at least 5 days. The knockout cell pools were subjected to further experiments, and the knockout efficiencies were examined by immunoblotting analysis.

### Mass spectrometry

HEK293T cells (1 × 10^8^) were transfected with FLAG-tagged hcGAS and PPP6C, or transfected with FLAG-tagged hcGAS and treated with OA (100 nmol/L). FLAG-tagged cGAS was immunoprecipitated and desalted. MS analysis was performed as previously described by SpecAlly (Wuhan) Life Science and Technology Company (Shang et al., 2018). Briefly, 50 µL SDC buffer (1% SDC, 100 mmol/L Tris-HCl, pH 8.5, 10 mmol/L TCEP, 40 mmol/L CAA) was added to the beads and heated to 95 °C for 10 min. The reaction was repeated and a total of 100 µL SDC reaction buffer was collected. Collected buffer was diluted with 100 µL H_2_O. One µg trypsin was added for overnight digestion at 37 °C. The next day, equal volume of 1% formic acid/ethyl acetate was used to stop the digestion. After centrifugation (12,000 ×*g*, 1 min), the supernatant was subjected to peptide purification using SDB desalting column. The peptide eluate was vacuum dried. LC-MS/MS data acquisition was carried out on a trapped ion mobility LC-MS/MS mass spectrometer (timsTOF Pro, BRUKER) equipped with a nanospray source. Peptides were fractionated in a C18 analytical column (75 µm × 250 mm, 1.6 µm particle size, 100 Å pore size, ionopticks). Mobile phase A (99.9% H_2_O, 0.1% formic acid) and mobile phase B (99.9% ACN, 0.1% formic acid) were used to establish a 75 min gradient, which comprised of: 0 min in 2% B, 50 min of 5%–23% B, 10 min of 23%–35% B, 5 min of 35%–90% B, 90% B for 10 min. A constant flow rate was set at 300 nL/min. For PEASF mode analysis, each scan cycle consisted of 1.11 second, in which 0.1 second for MS1 and the rest for MS2. Raw data from timsTOF Pro were analyzed with PEAKS Studio (V10) using the following parameters: database: Homo sapiens; Parent Mass Error Tolerance: 20ppm;Fragment Mass Error Tolerance: 0.05Da; Precursor Mass Search Type: monoisotopic; Enzyme: Trypsin; Max Missed Cleavages: 3; Fixed Modifications: Carbamidomethylation; Variable Modifications: Acetylation(Protein N-term), Oxidation(M); Max Variable PTM Per Peptide: 3. Search results were filtered with FDR threshold at 1% and unique peptide greater than 1.

### Preparation of recombinant cGAS^S420^ or cGAS^pS420^ proteins

The preparation of recombinant proteins including unnatural amino acids was described previously (Pirman et al., 2015). Briefly, SBP-tagged mcGAS cDNA in which Ser420 (AGC) codon is replaced with pSer420 (UAG) amber codon was constructed into the GFP E17TAG plasmid. The plasmids, which expresses SBP-mcGAS-420 (UAG) and OTSk or SBP-mcGAS-420 (UAG) and SupD were transformed into *E*. *coli* C321.ΔA.ΔserB strain. The transformed *E*. *coli* were grown overnight in LB medium supplemented with 0.08% glucose, 25 µg/mL kanamycin and 100 µg/mL ampicillin (LB-GKA medium) at 30 °C. Fifty milliliter overnight cultured bacteria were then inoculated into 500 mL LB-GKA medium supplemented with 2 mmol/L phosphoserine for further culturing. When OD600 of the culture reached 0.6–0.8 (normally in about 2 h), the bacteria were further treated with 1 mmol/L IPTG (for SEPOTS induction) and 100 ng/mL anhydrotetracycline (ATC, for mcGAS induction) at 30 °C for 24 h. The bacteria were than harvested and lysed. The recombinant cGAS^S420^ or cGAS^pS420^ was purified with Streptavidin Sepharose High Performance affinity resin (GE Healthcare) according to the manufacturer’s protocol. The purified proteins were separated by 10% SDS-PAGE and stained with Colloidal Blue Staining Kit (Invitrogen) according to the manufacturer’s protocol.

### Statistics

Data were analyzed using a Student’s unpaired *t*-test, multiple *t*-test or two-way ANOVA with Prism GRAGHPAD 7. For the mouse survival study, Kaplan-Meier survival curves were generated and analyzed by Log-Rank test. The number of asterisks represents the degree of significance with respect to *P* values, with the latter presented within each figure or figure legend.

## Abbreviations

BMDMs: bone marrow-derived macrophages
BMDCs: bone marrow-derived dendritic cells
cGAMP: cyclic GMP-AMP
CRISPR: clustered regularly interspaced short palindromic repeats
HSV-1: herpes simplex virus type 1
HSV120: 120-mer DNA representing HSV-1 genome
hcGAS: human cGAMP synthase
IFNs: type I interferons
IRF3: interferon regulatory transcription factor 3
ISRE: interferon-stimulated response element
mcGAS: mouse cGAMP synthase
MITA: mediator of IRF3 activation
MLFs: murine lung fibroblasts
PAMP: pathogen associated molecular pattern
PRRs: pattern recognition receptors
PPP6C: protein phosphatase 6
SAVI: STING-associated vasculopathy with onset in infancy
Sep: phosphoserine
SeV: Sendai virus
SLE: systemic lupus erythematosus
STING: stimulator of interferon genes
TBK1: TANK-binding kinase 1
